# Supplementary material for: Patient and Healthcare Professional Reflections on Consenting for Extra Bone Marrow Samples to a Biobank for Research—A Qualitative Study
Source: Curr Oncol. 2025 Mar 19;32(3):179. doi: 10.3390/curroncol32030179 (PMC11941294; doi:10.3390/curroncol32030179)
Supplement: Supplementary file 1 [file curroncol-32-00179-s001.zip › Table S1 .pdf]

Table S1. GRIPP2-SF.

Section and topic	Item	Reported in Page No
1: Aim	Report the aim of PPI in the study	3
2: Method	Provide a clear description of the methods used for PPI in the study	3-4
3. Study results	Outcomes—Report the results of PPI in the study, including both positive and negative outcomes	4
4. Discussion and conclusions	Outcomes—Comment on the extent to which PPI influenced the study overall. Describe positive and negative effects	4
5. Reflections/critical perspective	Comment critically on the study, reflecting on the things that went well and those that did not, so others can learn from this experience	N/A
