# Supplementary material for: Patient and Healthcare Professional Reflections on Consenting for Extra Bone Marrow Samples to a Biobank for Research—A Qualitative Study
Source: Curr Oncol. 2025 Mar 19;32(3):179. doi: 10.3390/curroncol32030179 (PMC11941294; doi:10.3390/curroncol32030179)
Supplement: Supplementary file 1 [file curroncol-32-00179-s001.zip › Table S2 .pdf]

Table S2. Theme and example quotes.

Theme	Example quotes
1. Reflections on the consent process	
a. Preparation or awareness of research	1.1 "The only thing I find is having come from solid tumour cancers to hematology cancers, I find there's a bigger disconnect between clinical trials and the regular staff because the treatments are given in the CIU, they're not given in the medical daycare. Whereas in solid tumour the treatments are given in clinical trials, but also in the regular chemotherapy unit. So, there's a lot more, let's say sensitization to clinical trials and research and following protocols and having study quick guides, so you know what to expect with the study drug and all that kind of stuff. Whereas in hematology it's completely separate and so I find I wish it was more the other way." *Staff, interviewee #3*<br><br>1.2 "I feel that if the people have the information, it's because […] they are part of the research so I would feel comfortable getting the information from anyone who has that in their hands." *Patient, Interviewee #3*<br><br>1.3 "For me, I you know, as long as it's like, honestly, I'm thinking it's some healthcare professional asking I'm OK with that as long as they're legit" *Patient, interviewee #11*<br><br>1.4 "but when they schedule you for a bone marrow aspiration, whether they should be saying, oh, and they may ask you about a research study while you're at it. Which would lead me to say to the doctor 'what kind of research study and what's it all about?' And that individual will go. I don't know. I'm just supposed to ask you about that? They're putting the burden on somebody else to have a bunch of answers." *Patient, Interviewee #7*<br><br>1.5 "Acknowledge the person have a real conversation with them, like take a deep breath and realize that like and it's understandable that this happens. But for healthcare providers and researchers, this is your daily schtick. But for the person you're talking to, this is like not their daily schtick, and they really don't want it to be their daily schtick." Patient, interviewee #20
b. Logistical aspects	1.6 "I would definitely recommend mentioning it when you're clothed, not in some hospital gown sitting like in a in a less of an imbalance sort of conversation situation for sure." *Patient, interviewee #20*

	1.7 "Well, it was easy for me. Well, because I was already in the hospital, I didn't have to get in my car and go anywhere. I didn't have to schedule an appointment. Go in my car, go to a new building. A new you know place. So, it was. I just had to lie there it was quite I it didn't. You know, I didn't have to go out of my way for it at all." Patient, interviewee #4
c. Having support	1.8 SN: Where would you say her biggest help or influence was? Was it around sort of information. Or was it that sort of practical things in terms of what you mentioned the few?<br><br>"Comfort. News. Uh, bringing information to me. Uh, talking to me a couple of times a day on the phone. Just to pass the time to give me something to do rather than just lying in bed during the period I was in the hospital. Umm yes. To listen to the nurse or whoever was talking to me, the pharmacist, whoever happened to come in while I was in the hospital and to just, I guess be the second the second set of ears to say no, that's not exactly what they said, or you missed this. Again, this was a period where I was a little hard of hearing before I had. I got hearing aids in now, so it was quite some quite easy for me to miss things going on. *Patient, interviewee #2*<br><br>1.9 "I think it's very fundamental to have someone to be your second ears and second eyes and second brain because you need someone to translate the information after because you're not in a situation where you can process but the, I know that more and people are alone now. And not having a family or people around. But at least make sure that someone will be able to tell the person after what was said because I remember better five years after than a day after when it happened. […] But having someone is fundamental." *Patient, Interviewee #13*
2. Decision-making and decisional needs	
a. Lack of deliberation	2.1 "It didn't bother me at the time. Like it just you know, if you want. Yeah, sure. You know it when you say no to something like that, or if one was the say no, you almost feel like ohh my God like is the next person is what? What happens if this could help the next person? I don't want to be that guy, right to say, you know what had I not or had I done it? Just do it." Patient, interviewee #11

	2.2 “yeah, it was easy. Especially like I don't know if it would have been as easy if it had required a different an additional biopsy like that's, you know, digging into your bones an extra time as not nothing. Umm, but yeah, it's super easy, right? I’d say yes.” Patient, interviewee #20<br><br>2.3 “I think majority of the time they [patients] pretty know what they want to say.” Staff, interviewee #3<br><br>2.4 “It's usually straightforward. The answer you're comes pretty quickly from both sides, yes or no.” Staff, interviewee #1
b. Proximal factors: immediate care, impact on health and family impact	2.5 “So that was part of my decision. One of my decisions to see if they could be a useful for or to see if it was in the family. You know, something like that.” Patient, interviewee #12<br><br>2.6 “Int1: Umm, sometimes. I guess like there's a chance of contaminations, which I was worried about and stuff like that, but not so much in the whole side effects of that.” Patient, interviewee #1
c. Proximal factors: Pain and sample collection	2.7 “I remember them telling me, OK, you know, OK, we've got whatever amount we need and OK, now we're just going to get a little bit more for the research amount. And I know they had a little bit of an issue getting a little bit more, I don't know if I don't, I couldn't see, I don't know. I didn't know what they were doing, but whatever they had to do to chisel into my hip or whatever it was they were doing there, they had to do a little bit more, I guess, to get more and probably that I knew that from the second time, but I didn't know the first time that they were actually maybe getting a little bit more.” *Patient, Interviewee #17*<br><br>2.8 “Because I say the biggest complaint from them would be like, well, “I don't want more pain” because there can be pain with drawing extra samples. It's not like we're going to a new position and I tell them that, but you know they do, they do need to know that with the drawing the samples like for just the diagnosis, there can be some of pain associated with it, so they get a little, they get nervous about extra pain and so, but then at the same time they may be willing to, you know, do something for research and at that time, “I'll tell them, well, we'll do the marrow, and once I'm done my samples, I'll ask you ‘biobank, OK?”, if you say yes, we'll draw it.” *Staff, interviewee #5*<br><br>2.9 “I understood the procedure, I knew that it was important to do this at this time, but there was really scared about the, the pain that I could the I could have after the procedure, yes, but it did not happen with the second

	one and the third one because I knew everything before. It was really OK, the only thing it was like the fact that I was scared about the pain, […]" *Patient, interviewee #22*
d. Distal factors: Helping others and helping themselves	2.10 "Gosh, you want to be as positive as possible and so, you know, I didn't know if I was going to make it through it. And I thought, well, if I make it through, it'll help me. And even if I don't make it through, it will help me. You know it'll help someone at some point and make all this stuff curable, right? I mean that I think that's something that someone could have used, you know, that language to say, you know, look at breast cancer, it's very much a manageable curable cancer, right? And boy, wouldn't it be nice if BLL slipped into that category as well and with research, that's what's going to happen and maybe that has already started to happen with me. I mean, I, you know, with the four rounds, I would have been dead in each round if I didn't have advanced care, right?" Patient, interviewee #X<br><br>2.11 "When we present research as being the tools that may help you and may help others after and how it will help eventually. And when you're sick, you just want to. And to feel that you're part of something and give a sense to this illness you have. So, when it is presented as research is for the help of others, and of maybe even yourself. It does give a sense to all that pain." Patient, interviewee #13<br><br>2.12 "Or regarding research, I think you must be prouder at the Ottawa hospital. Of what you're doing and. Show it like I was so happy to be part and to receive all this high level of treatments. And this quality of treatments that I would have been happy to see it on all the walls and that it would have been even more obvious that it's a hospital that is making research and that research is a part of everything that Ottawa Hospital has done, not aside the treatments, but in every little thing that are made. Uh, so when you feel like you have, it's not obvious, it's. It's less tempting to say that you want to be part of this things, but when it's something so obvious that the research and the Ottawa Hospital are one and it's not confronting, you don't even have to make the decision of do I want to be part of it or not? Because it's obvious." *Patient, interviewee #13*<br><br>2.13 "And if you follow the bouncing ball of like the of how much research went into me being alive. Today, it's pretty compelling to want to give tissue to make it better. [...] But to me, I just feel like as the beneficiary of so much research over years and years and like, I don't even know the amount of dollars. [...] if something that I can do which is like they're literally in there anyway, it takes us take the sample and pass it on and it can help streamline the process for patients and doctors in the system going forward. Then like I think that's a really compelling thing and I was I was quick to say yes to anything research related." *Patient, interviewee #20*

e. Trust and reciprocity	2.14 "I just want to say that when I was asked and I never, I would not have hesitated though, and I think that the doctor [name], Dr [name] and all them in there and let me just tell you, I have a respect for every one of them and I hope this government, you know, instead of doing cutbacks, cuts and doing things, you know, I would do it the other way that to give them more to support more than to be better and stronger." *Patient, interviewee #14*<br><br>2.15 "Uh, the organization right the way that that the hospital team were organized, regardless of who was coming through the door. My door and at the hospital knew exactly my file, so it's not. I was not a number. I was [NAME] […] and they knew everything about what they had to perform for me and that brought me a lot of confidence because I said I'm, I'm just going out, I can close my eyes and I'll do whatever you ask me to do because I feel totally confident in in the process." *Patient, interviewee #3*<br><br>2.16 "[…], it's really nice to try to just have that human touch as well for a few minutes before it. It's like say, wow, you know, are you OK? How are you doing? Like, don't have to talk to me if you don't want to. But like I see you acknowledge the crap that you are going through right now and need to and then maybe get to the question about can we have your tissue for research?" *Patient, interviewee #20*
3. Information needs and preferences.	
a. Practical information: pain and processes	3.1 "You know, if you're, if you're going to take 10 mL of blood or 20 mL or one vial of blood or two vials of blood doesn't matter if it. If it involves a separate insertion into your back, maybe, I don't think I'd mind doing it once, but if it was more than once and how much do you need and why do you need it? I would want to know a little bit more about it, but again, if you can just take twice as much whenever you're doing it for a clinical test, it's not an issue for me." Patient, interviewee #2<br><br>3.2 "I think all they really said was just kind of like while they're in there, they're just going to take an extra sample. And that was kind of all I needed to know. I kind of, I knew what if I hadn't gotten the bone marrow biopsy done before, maybe like, kind of like just a general going through the process. But I knew the process, so it was just kind of, yeah, they're just we're just going to take another sample and then that's all I need to know, really." Patient, interviewee #9<br><br>3.4 "So reinforcing that that it's not extra, extra invasion, I guess. Is important and that seems to reassure people and that it's only if we can, we're not going to, you know, put you through extra pain and stuff for nothing. So

	that part seems to help. But I think if we explained more what the purpose is and the end goal then I think people would more readily say yes." *Staff, interviewee #1*<br><br>3.5 "I had heard that bone marrow samples were a painful process. So, I had expected this pain, but to later find out that we'll just give you more anaesthetic. And then that wasn't painful. I mean that there was a bit of an ache, but you got an ache from a vaccine in your arm, you know? So, it's like, well, yeah, whatever you can deal with that, it's kind of a dull pain in your hip going forward. So anyway, that that's the biggest problem I would say." *Patient, interviewee #18*
b. Contextual information: What research is done and why?	3.6 "What are we looking at with the biobank? Like what sort of research do we do on these samples? You know what's its purpose? Kind of thing as opposed to like the, I haven't read it fully, the sheet that we give, or the papers that we give, but it seems a little generic in terms of like research in itself, as opposed to like having something that actually says what I told them, when I consented them and how that would benefit hematology and future, whatever we're doing for the biobank as a whole." *Staff, interviewee #5*<br><br>3.7 "I think that would be cool. Yeah. And then don't get me wrong, I could have maybe gone something and just completely forgot about it, but yeah, I think it would be cool to kind of learn about what the research is doing and kind of what my sample is helping with.<br><br>3.8 "Also, I think is like it goes back to what I was saying about making it really clear that, but this is a really highly valuable thing to keep on studying these cells and these people and what existed like if you have the pre chemo my pre chemo cells and now you see the outcome and you know what exactly what it took like that's really informative and you get like yeah. So, I think that that is something to think about for sure." Patient, interviewee #20
c. Contextual information: People & privacy	3.9 "Confidentiality. Privacy. I think you know, uh, making sure people feel comfortable about that aspect of it, I think would be really good and maybe also being really clear. I'm pretty sure that they're really strict rules about if this, like my sample, is tied to, I've consented to have it used for some slice of research, and if someone wanted to research this completely other thing, they wouldn't technically be allowed. You know, like it's there's they're tight controls on that stuff. So, I think making sure that people feel comfortable from that side, but from the identity security, genetic information, things like that. Yeah." *Patient, interviewee #20*<br><br>3.10 "Where are they going after? And for the use of who? Who will be allowed to use them? Maybe just knowing who who's involved in the study and the scope of the study, and you know, how, is it just the Ottawa hospital, is it the is it Canada wide, is it you know international?" *Patient, interviewee #13*

d. Information about research (for the staff)	3.11 “It was only the one time that caught me a bit off guard that didn't have the answer to, and then I felt like not informed because like, why am I asking a patient for the sample if I don't really know where it's going.” Staff, interviewee #3<br><br>3.12 “So if I had to give a recommendation going forward, it would be for there to be like a maybe a couple of like lunch and learn sessions where you go [the] over consent process and the biobank and the goals of the biobank and what they're consenting to so that we can help support the process more.” Staff, interviewee #1
e. Format and the use of information (how to present information)	3.13 “For like learning about the research just in general, I think like a link to a website, or something would be good because then you can kind of save it on your browser and go back to it and not worry about losing a piece of paper which I'm very bad at." Patient, interviewee #9<br><br>3.14 “For me, the paperwork is fine because anyway I got a bunch of paperwork from the hospital when I was in. Uh, so I would like to have a folder and keep all this paper altogether. Well, if, uh, the information comes from a website or somewhere else, I still need a paper to give me the link, right? I mean, anyway, I'll have a bunch of paper. I still have this folder in my drawer. All the information from the hospital, I mean paper is OK for me. If you escape you, you get more information from our website. Cool, but the link will be on the paper so I can get there, yeah.” *Patient, Interviewee #8*<br><br>3.15 “Umm, they're kind of just like, Yep, Yep, Yep. Just go with it. Right. I think the reading information is nice because they can just sit and read it after. Once everything has calmed down.” *Staff, interviewee #3*<br><br>3.16 “Would have been interesting to have like a single sheet little handout that says “This is why we're taking it. This is what we're doing” so I can hand that to my partner. Say “I'm doing this,” “oh that's kind of cool” you know,” *Patient, Interviewee #21*<br><br>3.17 “maybe seeing having a physical piece of paper to hold. And like, if you're sitting in the hospital anyway and it's sitting beside your bed and you're whatever, just leafing through all the materials. I remember having booklets about various things. Umm, maybe some, maybe some actual paper copy stuff would be helpful. Just because in the moment? Yeah, stuff goes in and may or may not stick, so having a piece of paper to look back at and even, you know, write notes on or something like that, or a contact number for someone to call and ask like the questions too, could be good to...” *Patient, Interviewee #20*
